# Novel GxE effects and resilience: A case:control longitudinal study of psychosocial stress with war-affected youth

**DOI:** 10.1371/journal.pone.0266509

**Published:** 2022-04-04

**Authors:** Connie J. Mulligan, Christopher J. Clukay, Anthony Matarazzo, Kristin Hadfield, Lisa Nevell, Rana Dajani, Catherine Panter-Brick

**Affiliations:** 1 Department of Anthropology, University of Florida, Gainesville, Florida, United States of America; 2 Genetics Institute, University of Florida, Gainesville, Florida, United States of America; 3 School of Psychology, Trinity College Dublin, Dublin, Ireland; 4 Trinity Centre for Global Health, Trinity College Dublin, Dublin, Ireland; 5 Department of Biology and Biotechnology, Faculty of Science, The Hashemite University, Zarqa, Jordan; 6 Department of Anthropology, Yale University, New Haven, Connecticut, United States of America; 7 Jackson Institute of Global Affairs, Yale University, New Haven, Connecticut, United States of America; UCSI University, MALAYSIA

## Abstract

Responses to early life adversity differ greatly across individuals. Elucidating which factors underlie this variation can help us better understand how to improve health trajectories. Here we used a case:control study of refugee and non-refugee youth, differentially exposed to war-related trauma, to investigate the effects of genetics and psychosocial environment on response to trauma. We investigated genetic variants in two genes (serotonin transporter, *5-HTT*, and catechol-O-methyltransferase, *COMT*) that have been implicated in response to trauma. We collected buccal samples and survey data from 417 Syrian refugee and 306 Jordanian non-refugee youth who were enrolled in a randomized controlled trial to evaluate a mental health-focused intervention. Measures of lifetime trauma exposure, resilience, and six mental health and psychosocial stress outcomes were collected at three time points: baseline, ~13 weeks, and ~48 weeks. We used multilevel models to identify gene x environment (GxE) interactions and direct effects of the genetic variants in association with the six outcome measures over time. We did not identify any interactions with trauma exposure, but we did identify GxE interactions with both genes and resilience; 1) individuals with high expression (HE) variants of 5-HTTLPR and high levels of resilience had the lowest levels of perceived stress and 2) individuals homozygous for the Val variant of *COMT* with high levels of resilience showed stable levels of post-traumatic stress symptoms. We also identified a direct protective effect of 5-HTTLPR HE homozygotes on perceived insecurity. Our results point to novel interactions between the protective effects of genetic variants and resilience, lending support to ideas of differential susceptibility and altered stress reactivity in a cohort of war-affected adolescents.

## Introduction

Responses to childhood trauma and early life adversity differ greatly across individuals, spurring debate about the role of genetic and environmental factors. This debate is important as it may help shed light on how to improve health outcomes of children and adolescents who experience trauma. Individuals who experience childhood trauma are at increased risk for a wide range of psychological, physiological, cognitive, and behavioral conditions, which are likely to be of clinical and public health relevance [1–3]. Refugee populations are deemed particularly vulnerable to the risks of war-related trauma, although there is no one-to-one association between risk exposures and mental health outcomes given differences in processes of recovery, resilience, and supportive social networks [4]. Understanding how war-related trauma affects childhood health and development is critical since over half of the 26 million refugees worldwide are children and adolescents [5]. Calls have been made for research to evaluate the extent to which scientific work can help develop interventions or changes to the environment that reduce stress, improve mental health, and develop resilience in affected populations [6].

Traumatic events can be a single incident or repeated and ongoing trauma, and can include physical or emotional or sexual abuse, neglect, domestic violence, natural disasters, mass shootings, and war trauma. Response to all types of trauma is a complex phenotype that involves genetic and environmental factors, as well as interactions between the two. Accordingly, *differences* in response to trauma reflect variation in underlying genetic variants and environmental exposures. A genetic variant may act *directly* on an outcome phenotype (in concert with other factors) or may *interact* with an environmental exposure to influence the outcome. Gene x environment (GxE) effects are statistical interactions that model the way in which genetic variants may influence the effect of environmental factors on a phenotype and are particularly important when studying complex phenotypes like response to trauma.

Boyce and Ellis proposed that heightened stress reactivity might indicate an increased ‘biological sensitivity to context’ with the potential for more negative outcomes under adverse conditions but more positive outcomes with a protective or supportive environment [7]. An adaptive response to trauma may be both facilitated by, and result in, increased sensitivity and reactivity to a range of environmental factors [8]. In a more explicit description of how genetics and environment may interact, Belsky and Pluess [9, 10] proposed that genetic variants do not function just to increase the risk of negative outcomes, but instead function as ‘differential susceptibility’ variants that increase susceptibility or sensitivity to multiple environmental influences, such that carriers are more sensitive to both positive and negative factors. Their ‘vantage sensitivity’ model explains that some individuals are more sensitive to, and therefore better able to benefit from, positive environmental factors [11, 12].

The polymorphic region of the serotonin transporter (5-HTTLPR) has been proposed as a differential susceptibility variant in which individuals who carry the susceptibility variant show variation in adult empathy traits in response to different levels of childhood trauma [13]. The serotonin transporter protein helps transport serotonin from synapses to presynaptic neurons and is the target of many antidepressant and antianxiety medications. 5-HTTLPR is a functional polymorphism located in the promoter region on chromosome 17 and consists of 11–16 repeat units and a single nucleotide polymorphism (SNP, rs25531). The repeat polymorphism and A/G SNP create multiple variants that are typically classified as low and high expression (LE and HE) variants. LE variants have lower serotonin reuptake activity and higher levels of intrasynaptic serotonin compared to HE variants [14, 15]. A landmark study in 2003 reported that the LE variant moderated the influence of stressful events on depression [16]. This GxE effect of the LE variant and life stress has been replicated in additional studies of adults [17] as well as children and adolescents [18]. However, it is worth noting that not all studies have replicated the GxE effect and a meta-analysis found no evidence of an interaction between stress and 5-HTTLPR on risk of depression [19]. Recent studies have found an association between 5-HTTLPR and resilience [20, 21], suggesting that replication of candidate genes may be complicated when comparing studies that test different adverse and protective factors. Direct effects of the 5-HTTLPR polymorphism have also been identified, such as a study of children and adolescents exposed to the 2008 Wenchuan Earthquake that reported LE homozygotes had higher initial PTSD symptom severity but faster recovery rates [22] and a study of combat-exposed veterans in which LE carriers showed increased risk of PTSD but only in the Black study population [23]. Other studies report that carriers of the HE variant show improved response with antidepressant medication [24], suggesting that the polymorphism may play a causative role in response to stress and related psychiatric disorders.

Another gene that has been studied for its role in moderating response to stress and related anxiety and mental health disorders is catechol-O-methyltransferase (*COMT*) [25]. *COMT* encodes a protein involved in the degradation of catecholamine neurotransmitters like dopamine, epinephrine, and norepinephrine. The Val158Met substitution is a functional polymorphism in which the Met allele has 3-4-fold reduced COMT activity resulting in lower levels of neurotransmitter degradation and higher levels of transmitter availability [26]. The ancestral Val allele has been reported in control populations at highest frequencies in Asians (69–80%) and lower frequencies in Caucasians (46–60%), North Africans (50–80%), and Arabs (44–50%) [27–30]. *COMT* is located in a region of chromosome 22 that was implicated in the pathogenesis of schizophrenia in early linkage analysis studies so many *COMT* studies have focused on its association with schizophrenia and related risk factors like stress and drug use [31, 32]. Early studies reported both the Val and Met alleles as risk alleles depending on the population and health outcome studied [e.g. 31, 33]. More recent studies have identified GxE interactions with childhood trauma and early life adversity on psychotic experiences and risky behaviors [34–36]. Lovallo et al. [35, 36] reported that Met carriers had progressively smaller cortisol responses with greater adversity and increased risk of drinking and drug-use behaviors whereas Val homozygotes showed blunted cortisol responses and little effect on risky behaviors. There is evidence that the Val allele may function as a differential susceptibility variant based on a report that Val homozygotes, but not Met homozygotes, showed a higher startle response with greater childhood trauma [37]. However, not all studies have been able to replicate these GxE interactions [reviewed in 32], and such interactions may be developmentally dependent and vary by age and sex.

In the current study, we investigated the effects of genetics and psychosocial environment on response to trauma in a case:control study of Syrian refugee and Jordanian non-refugee youth who were differentially exposed to war-related trauma. We assayed variants in two genes; 5-HTTLPR and an associated A/G SNP, rs25531, in the serotonin transporter gene 5-HTT and the Val158Met SNP in *COMT*. In terms of environmental factors, we collected data on number of lifetime traumatic events and levels of resilience in order to investigate adverse and protective psychosocial factors, respectively. We directly measured resilience using a scale specifically developed for youth in adversity to assess culturally-relevant resources at the individual, community, and contextual levels [38, 39]. In order to gauge response to trauma, we measured six mental health and psychosocial stress outcomes. Finally, we tested for direct and interactive (i.e. GxE) associations with the genetic variants, and trauma or resilience, on year-long trajectories of the outcome measures. We predicted both direct genetic effects (that influence behavior directly, in concert with other factors) and GxE effects (that influence environmental effects on behavior). Specifically, because both 5-HTTLPR and *COMT* play a key role in the stress response and resilience [40], we predicted that the tested genetic variants would show direct protective effects, and also GxE effects with resilience, in association with improved scores on a subset of the outcome measures focused on psychosocial stress.

## Materials and methods

### Study design

We used a case:control study of refugee and non-refugee youth, with higher and lower levels of lifetime traumatic events, respectively, in order to investigate the effects of genetics and psychosocial environment on response to trauma. The study design and sample collection were described in previous publications that report on the effectiveness of the stress attunement intervention [41]. Briefly, buccal samples and survey data were collected from a gender-balanced sample of 417 Syrian refugee and 306 Jordanian non-refugee youth, aged 12–18 years old and living in the same city neighborhoods within Jordan. Syrian and Jordanian youth were enrolled in a randomized controlled trial testing the impacts of a stress attunement intervention program (Advancing Adolescents, an 8-week program of structured activities) delivered by Mercy Corps to children and adolescents affected by the Syrian crisis. There were no significant interactions between the intervention and the genetic variants in association with the outcome measures so in the current study the intervention was analyzed as a covariate only. Data were collected at baseline (T1), ~week 13 post-intervention (T2), and ~week 48 follow-up (T3). There were 538 participants at T2 (26% attrition) and 273 participants at T3 (62% attrition), reflecting a highly mobile population in which only 0.018% and 8% of participants declined to participate at T2 and T3, respectively, and the remaining participants were unreachable by phone or had moved away/busy with school/at work/etc. In the first wave of study (March-June 2015, *n* = 183), participants were quasi-randomized into treatment and control groups, and in the second wave (September 2015-February 2016, *n* = 540), participants were fully randomly allocated to treatment or wait-listed control. There were no significant differences in the main independent variables (5-*HTTLPR* and *COMT* variants, trauma exposure), enabling us to combine the two waves for a total sample of 723 adolescents.

The study received approval from the Prime Minister’s Office of Jordan and ethical approval from Yale University (IRB ID 1502015359). Written informed consent to participate was given by parents or guardians, and verbal assent was obtained from each participant, after Arabic-language information sessions had been held with families at public community meetings. Capacity to provide consent by participants was evaluated by Mercy Corps program officers, following culturally-relevant consent procedures developed with families and community leaders. All procedures for determining capacity to consent and for obtaining written and verbal consent were formally approved by both IRBs. A copy of the PLOS questionnaire on inclusivity in global research is available as S1 File. This study is registered under ClinicalTrials.gov ID NCT03012451.

### Trauma, resilience, mental health and psychosocial stress measures

Lifetime trauma exposure was assessed with the Traumatic Events Checklist, adapted for use with adolescents in conflict regions [42]. Resilience was measured using the 12-item Child and Youth Resilience Measure (CYRM) as a self-reported scale specifically developed to assess resilience in youth living in adversity [38]. This measure of resilience is situated within a socioecological framework [38, 39] and assesses protective factors at individual, relational, and contextual levels, rather than treating resilience as an internal trait or using better-than-expected functioning as a proxy for resilience. The resilience measure was developed during this study so it was only collected in the second wave of survey for 540 individuals.

We tested six mental health and psychosocial stress outcomes, using three international (PSS, SDQ, CRIES-8) and three regional (HD, HI, AYMH) questionnaires. The Perceived Stress Scale (PSS, 14 items) assessed perceived psychosocial stress, developed in Western settings and validated in Jordan [43, 44]. The Human Distress (HD, 12 items) and Human Insecurity (HI, 10 items) scales, capturing symptoms of distress/stress and fear/insecurity, respectively, were specifically developed for use in conflict settings in the Middle East region [45]. The Arab Youth Mental Health (AYMH, 21 items) scale was specifically developed to screen for depression and anxiety in Arab youth [46]. The Strength and Difficulties Questionnaire (SDQ, 25 items) is a widely-used psychometric instrument to assess behavioral and emotional mental health difficulties [47–49]. Finally, the Children’s Revised Impact of Event Scale (CRIES-8, 8 items) measures symptoms of posttraumatic stress [50, 51].

### Buccal sample collection and DNA extractions

Buccal samples were collected using Transport Swabs (APCO Laboratory Consumable Plastic, Jordan) or DNA Buccal Swabs (Isohelix, United Kingdom). After rinsing their mouths with water, participants brushed both sides of their mouth with the collection swab for up to 30 seconds. DNA was extracted from Transport Swabs using the Qiagen DNA Investigator Kit (Qiagen, USA) and from DNA Buccal Swabs using the Xtreme DNA Isolation Kit (Isohelix, United Kingdom). DNA extractions were performed according to manufacturer’s recommendations with the exception that the AW2 wash was performed twice for swabs extracted using the Qiagen kit. DNA samples were genotyped at least two times for each variant and discrepancies were resolved by genotyping a third time. All genotype frequencies were in Hardy-Weinberg equilibrium.

### Genotyping and allele coding

#### 5-HTTLPR

A repeat polymorphism and associated single nucleotide variant (rs25531) in the 5-HTTLPR of the serotonin gene (*5-HTT*) were assayed based on a modified protocol [16] using the GoTaq^®^ PCR Core System I (Promega, USA) and the following primer sequences: Forward 5’-ATGCCAGCACCTAACCCCTAATGT-3’, Reverse 5’-GGACCGCAAGGTGGGCGGGA-3’. The reaction mixture consisted of: 3μL genomic DNA, 1.25mM MgCl2, 10μL 5X Green GoTaq^®^ Flexi Buffer, 1.25U GoTaq DNA polymerase, 1μL 10mM nucleotide mix, and 0.6μL of 10μM forward and reverse primers in a final reaction volume of 50μL. This MgCl_2_ concentration is lower than in many standard master mixes, consistent with previous literature regarding the need for a lower MgCl_2_ concentration [52]. The PCR profile was as follows: 95°C for 5 min followed by 34 cycles of 94°C for 30 sec, 66°C for 30 sec, and 72°C for 30 sec, followed by a final 3 min step at 72°C.

An A/G polymorphism (rs25531) present in L alleles was assayed in all amplification products by digestion with 24U of *Msp*I (New England Biolabs) overnight at 37°C based on previous studies [53]. Digested products were electrophoresed on 3% agarose gels using Agarose SFR^™^ (VWR Life Science, USA) for 3 hours at 80V.

**Fig 1:**
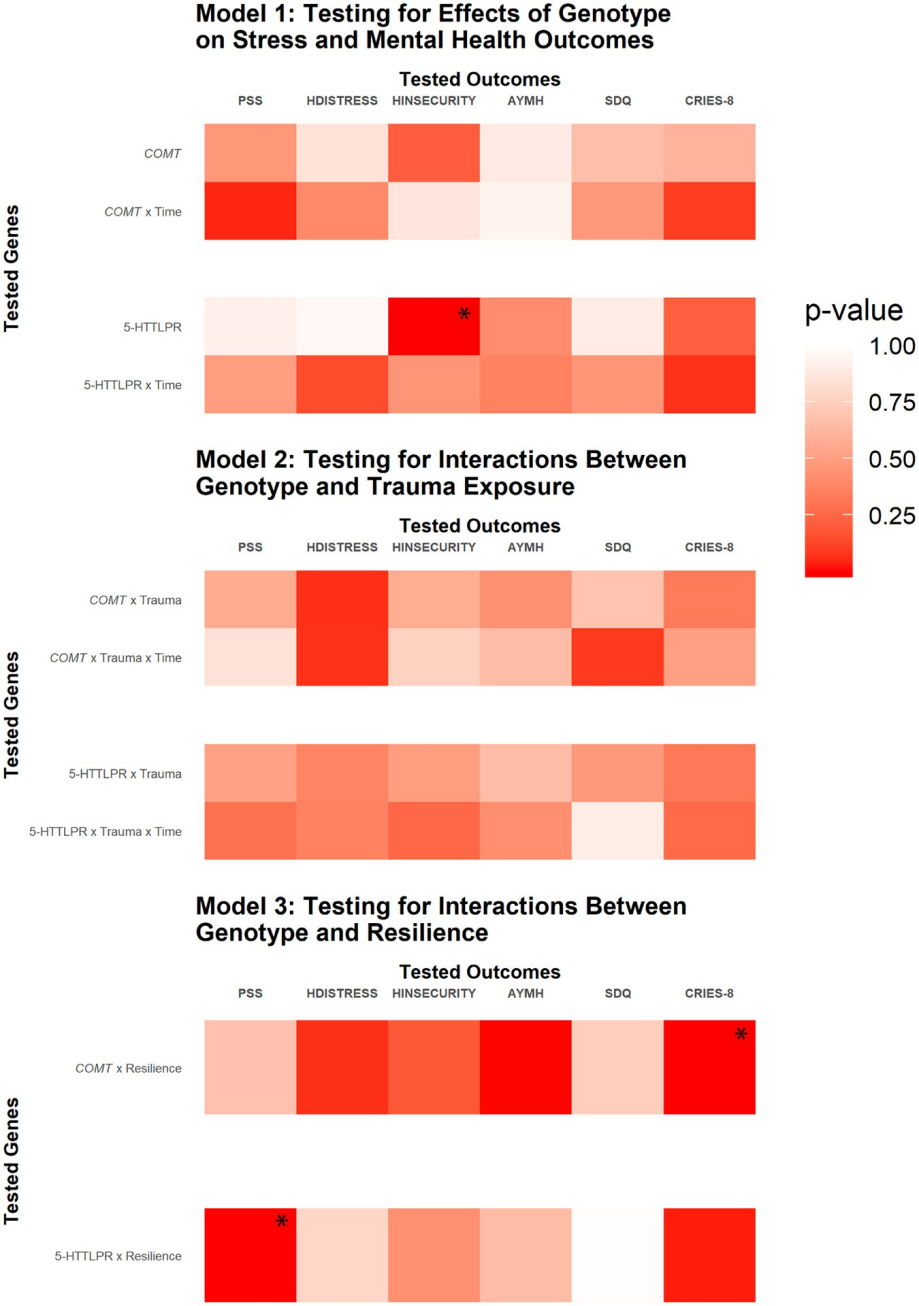
Heatmap summary of results from three multilevel models testing for direct genetic/trauma interaction/resilience interaction effects on six mental health and psychosocial stress outcomes. *COMT* and 5-HTTLPR genotypes (listed on the left) were tested for direct effects (Model 1), interactions with trauma (Model 2), and interactions with resilience (Model 3) on the six outcome measures (listed across the top of each figure). For each model and gene, intercept effects are listed first followed by slope (x Time) effects [except for Model 3 in which intercept and slope effects are combined into a single term because one of the independent variables (resilience) varies over time]. Darker red color indicates a smaller p-value and the three significant results (after multiple testing correction) are marked with an asterisk.

**Fig 2:**
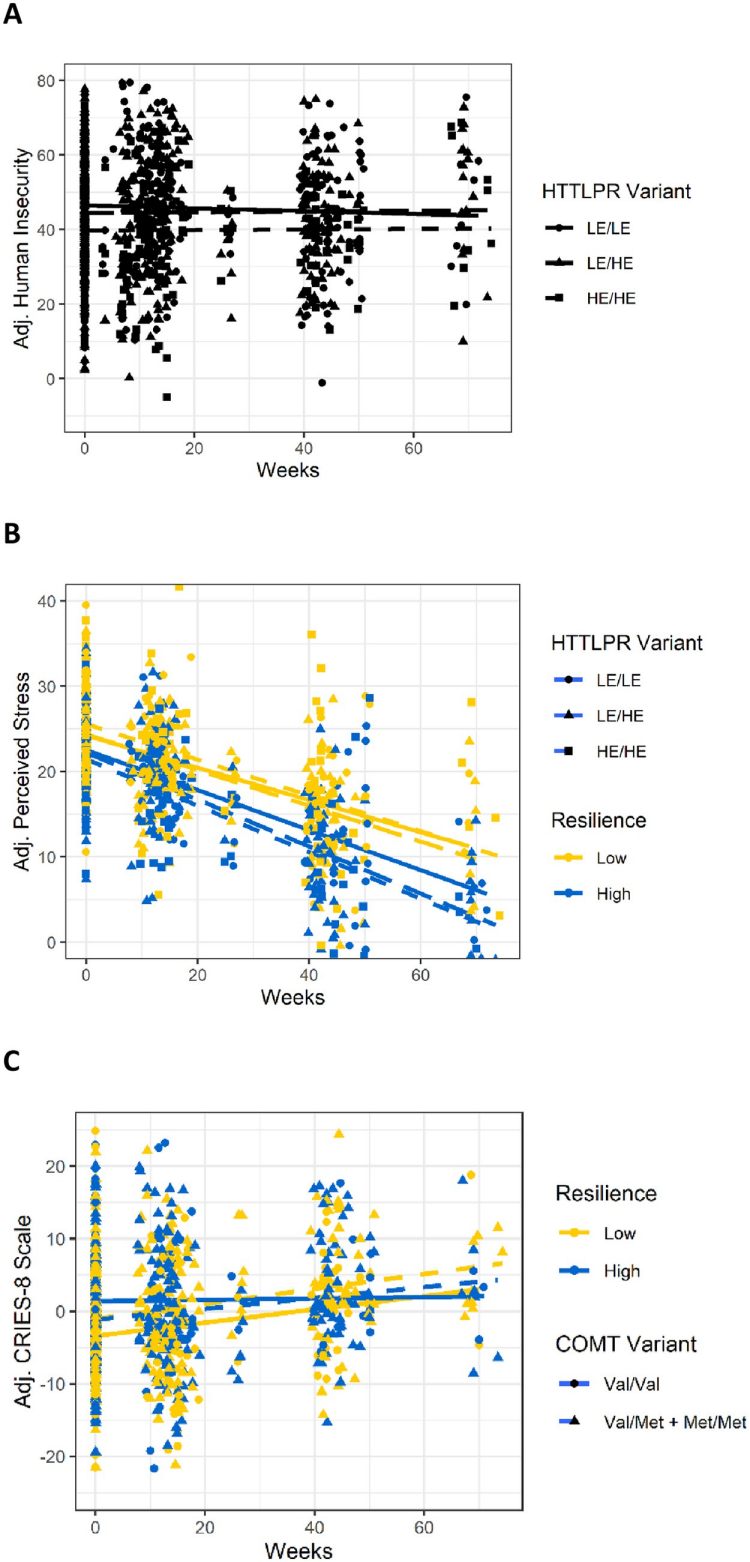
Partial effect plots of mental health and psychosocial stress outcomes over time by genetic variant. Outcome scores were adjusted for the effects of all covariates in the model and plotted (Y axis) for each participant at all time points (X axis). In B and C, resilience was dichotomized around the median (median = 51) purely for visualization purposes but was treated as a continuous measure in multilevel models. A. Human Insecurity (HI) was plotted over time by 5-HTTLPR variant. 5-HTTLPR HE homozygotes (dotted line) had lower levels of HI relative to LE carriers. B. Perceived psychosocial stress (PSS) was plotted over time by 5-HTTLPR variant and resilience levels. Partial effect plot lines were fitted for the six categories combining 5-HTTLPR genotype (LE/LE, LE/HE, HE/HE) and resilience (low and high). Individuals with high levels of resilience (blue lines) had lower average levels of PSS at all time points. HE carriers with high levels of resilience (blue dotted and dashed lines) had the sharpest average decline in PSS. C. Post-traumatic stress symptoms (CRIES-8) plotted over time by *COMT* variant and resilience levels. Partial effect plot lines were fitted for the four categories combining *COMT* genotype (Val/Val or Met carriers) and resilience (low and high). Val homozygotes with high levels of resilience (blue solid line) showed stable post-traumatic stress symptoms over time. All other individuals (Val homozygotes with low resilience and Met carriers with high and low resilience) showed an increase in post-traumatic stress symptoms over time.

Combined repeat and A/G polymorphisms were separated into two categories of high and low expression variants based on the literature [15]. All XS and S variants (≤ 14 repeats), as well as L variants containing a G at rs25531 (16 repeats + G), were coded as low expression (LE, comparable to S alleles in previous studies) and L variants containing an A at rs25531 were coded as high expression (HE, comparable to L alleles in previous studies) [14, 53]. Due to the rarity of XL variants (>16 repeats) and the lack of consensus on their expression levels [14, 15], the four individuals carrying XL variants were excluded from analysis. HE and LE variants were analyzed in two ways, by dosage effect or genotype (HE/HE vs HE/LE vs LE/LE) and by HE homozygote vs Other (HE/HE vs HE/LE + LE/LE)–results by dosage effect coding are presented in Figs 1 and 2, but results are only discussed if they were robust to both codings.

#### COMT

The rs4680 polymorphism of *COMT* was assayed using a predesigned TaqMan Genotyping Assay (Assay ID: C__25746809_50; Applied Biosystems, USA). Amplification was performed using manufacturer recommended conditions for a 96-well plate. Dilute samples of 5 ng of DNA in 11.25 μL dH_2_O were mixed with 12.5μL of 2X TaqMan^®^ Genotyping Master Mix (Applied Biosystems, USA) and 1.25μL of 20X TaqMan^®^ Drug Metabolism Genotyping Assay Mix (Applied Biosystems, USA) for a total reaction volume of 25μL. The cycling profile was: 95°C for 10 min followed by 50 cycles of 92°C for 15 sec and 60°C for 90 sec. Genotypes were determined after amplification using dye fluorescence intensities via the Bio-Rad CFX Manager with lab member and blank controls in triplicate as references. Individuals were coded as Val/Val homozygotes, Val/Met heterozygotes, and Met/Met homozygotes for analysis.

### Statistical analyses

We tested hypotheses that each genetic variant was associated with each of the six mental health and psychosocial stress outcomes, either directly or through interactions. To do so, we used multilevel models (time points nested within participants), or growth curve models, to test for effects of 5-HTTLPR or *COMT* genotype, directly or through statistical interactions with trauma exposure or resilience, on the six outcomes over the course of approximately one year. Multilevel models use all available data and are robust to partially missing data, yielding higher statistical power than linear mixed models and regression analyses, while still taking advantage of a longitudinal design [54, 55].

All statistical analyses were conducted in R [56]. Multilevel modeling was used to test for the significance of all associations using the nlme package [57]. A nested model structure was used in which timepoints (level one) were grouped within individuals (level two). Age, sex, collection site, and country of origin were included as time-invariant, fixed effect covariates. The intervention, administered between T1 and T2, was included as a time-varying covariate where all individuals were coded as 0 at T1 and those who participated in the intervention (i.e. the treatment group) were coded as 1 at T2 and T3. Consistent with our previous studies [41, 58–60], trauma was treated as time-invariant using trauma exposure data collected at T1 and was included as a covariate in the model that tested for interactions with resilience. For the second wave participants, in which the resilience measure was developed and collected, resilience was treated as a time-varying measure, meaning the value at each timepoint was included. Follow-up analyses were conducted in which kinship (measured as yes/no for siblings in the study sample) and time-in-Jordan (to reflect time since the stress exposure and measured at T1 just for Syrian refugees) were added to the above analyses as time-invariant, fixed effect covariates.

In order to test whether sufficient variation existed to use multilevel modeling, we tested for an association between time and each mental health and psychosocial stress outcome; all six outcomes (PSS, HD, HI, AYMH, SDQ and CRIES-8) had sufficient variation with time for both genes (*p* < 0.05). This finding remained true when testing the subset of individuals with resilience measures.

Three sets of models were run: Model 1 tested for direct genetic effects on the six outcome measures, Model 2 tested for a genetic interaction with trauma on the outcome measures, and Model 3 tested for a genetic interaction with resilience on the outcome measures. For each set of models, the base model included time and covariates for age, sex, collection site, country of origin, and intervention participation. Independent variables 5-HTTLPR, *COMT*, trauma exposure, and resilience were added to the base model to test for associations between the independent variables and each of the six outcome measures.

Significance tests for variables of interest were conducted by performing an ANOVA on nested models, one with the variable of interest and one without it. Time-invariant terms, like genotype and trauma exposure, have both intercept effect terms and slope effect terms whereas time-varying terms, like resilience, have slope and intercept effects combined into a single term. Significance after correction for multiple testing was calculated as *p* < 0.05 / [(2 loci x 6 outcome measures x 3 models (direct/trauma/resilience)] = 1.4 x 10^−3^.

In order to visualize the effects that we report, partial effect plots were constructed using the lme4, remef, and ggplot packages [61–63]. All plots were corrected for the same covariates (age, sex, collection site, country of origin, and intervention participation) that were included in the significance testing. For the partial effect plots, resilience scores were dichotomized at the median (median based on averages of all resilience measures for each individual = 51) to more clearly illustrate the effects (resilience scores were treated as continuous variables in the multilevel models and when testing for significance).

## Results

### Sample characteristics

Sample characteristics are shown in Table 1. Genetic data were collected for 415 male and 308 female participants at T1, with retention of 74% and 38% of participants at T2 and T3, respectively. Exposure to traumatic events was significantly higher for male relative to female participants, but levels of resilience resources were comparable by gender. In terms of the mental health and psychosocial stress outcomes, perceived stress (PSS), distress (HD), insecurity (HI), anxiety and depression (AYMH), and behavioral and emotional mental health (SDQ) symptoms were significantly higher in females than males. When comparing populations, Syrian refugees reported a significantly higher average of six lifetime traumatic events versus an average of one event reported by Jordanian non-refugees. They also reported significantly higher perceived stress, distress, insecurity, anxiety and depression, behavioral and emotional mental health symptoms, and much higher post-traumatic stress symptoms than Jordanian non-refugees, although all scores were relatively high reflecting the disadvantaged neighborhoods of both populations. Syrians averaged lower resilience scores than Jordanian counterparts. No differences in trauma exposure or resilience levels were detected between 5-HTTLPR genotypes or between *COMT* genotypes (*p* < 0.05, data not shown).

**Table 1:** Sample characteristics at baseline.

		Males	Females	Syrian refugees	Jordanian non-refugees
**Participants (#)**		415	308	417	306
**Age**^a^ **(years)**		14.1	14.7	14.3	14.5
		(1.67)	(1.79)	(1.85)	(1.58)
***COMT***	**Val/Val (#)**	107	78	102	83
	**Val/Met (#)**	207	152	208	151
	**Met/Met (#)**	99	77	107	69
**5-HTTLPR region of *5-HTT*/ *SLC6A4***	**High Expression (#)**	80	67	105	42
	**High-Low Expression Heterozygotes (#)**	173	118	185	106
	**Low Expression (#)**	104	83	117	70
**Lifetime Traumatic Events** ^a^ ^,^ ^b^		4.39	3.75	6.38	1.03
		(3.85)	(3.61)	(3.24)	(1.58)
**Child and Youth Resilience Measure**^b^^,^^c^ **(CYRM12)**		50.4	49.9	49.4	51.2
		(6.72)	(6.86)	(6.91)	(6.48)
**Perceived Stress Scale**^a^^,^^b^ **(PSS)**		26.7	28.7	28.6	26.0
		(5.71)	(6.61)	(5.85)	(6.33)
**Human Distress Scale**^a^^,^^b^ **(HDISTRESS)**		34.9	42.1	41.8	32.7
		(18.6)	(22.5)	(21.0)	(19.0)
**Human Insecurity Scale**^a^^,^^b^ **(HINSECURITY)**		62.9	67.7	67.8	61.0
		(21.6)	(20.5)	(19.7)	(22.5)
**Arab Youth Mental Health**^a^^,^^b^ **(AYMH)**		32.7	36.1	35.8	32.0
		(7.60)	(9.15)	(8.57)	(7.78)
**Strengths & Difficulties Questionnaire**^a^^,^^b^ **(SDQ)**		14.0	16.3	15.5	14.3
		(5.87)	(6.21)	(6.00)	(6.23)
**Children’s Revised Impact of Events**^b^ **(CRIES-8)**		13.3	13.6	19.4	5.27
		(12.3)	(13.0)	(10.9)	(9.89)

Unless otherwise stated, values are reported as ‘mean (SD)’ and are from surveys taken at baseline. For 5-HTTLPR, variants with ≤14 repeats or 16 repeats + G at rs25531 were classified as low expression (LE) and variants with 16 repeats + A at rs25531 were classified as high expression (HE).

^a^ Denotes a statistically significant difference between male and female participants (*p* < 0.05).

^b^ Denotes a statistically significant difference between Syrian and Jordanian participants (*p* < 0.05).

^c^ This measure was only collected with a subset of 311 male and 219 female participants.

### 5-HTTLPR—Direct genetic effects

When we tested for direct genetic effects (Fig 1, Model 1), we found that 5-HTTLPR was significantly associated with Human Insecurity (HI), even after correction for multiple testing (5-HTTLPR *p* = 5.0 x 10^−4^, S1 Table). A plot of 5-HTTLPR and HI shows that high expression (HE) homozygotes had lower levels of HI relative to low expression (LE) allele carriers (Fig 2A). No significant direct genetic effect associations of 5-HTTLPR and the other five outcome measures were identified (Fig 1, Model 1). In follow-up analyses that added measures of kinship and time-in-Jordan (just for Syrian refugees) as covariates, overall results were unchanged, *p* values generally decreased (9 out of 12 analyses), and 5-HTTLPR remained significantly associated with HI (5-HTTLPR *p* = 5.21 x 10^−5^, S2 Table).

### 5-HTTLPR—Interactive effects

Although previous studies have found GxE interactive effects between 5-HTTLPR and adversity [16–18], we did not identify significant interactive effects of 5-HTTLPR and trauma exposure after multi-testing correction (Fig 1, Model 2; S1 Table). When we tested for interactions between 5-HTTLPR and resilience (Fig 1, Model 3), we did identify a significant GxE interaction between 5-HTTLPR and resilience on perceived psychosocial stress (PSS) (5-HTTLPR x Resilience *p* = 7.6 x 10^−4^, S1 Table). A plot of 5-HTTLPR and PSS shows that individuals with high resilience had lower levels of PSS than individuals with low resilience (Fig 2B). The GxE effect of 5-HTTLPR and resilience was revealed within the group of high resilience individuals where the HE allele had a protective effect; among high resilience individuals, HE carriers had sharper declines in PSS relative to LE homozygotes (Fig 2B). The two results with 5-HTTLPR were robust to coding the variants as HE homozygotes vs LE carriers in addition to the dosage coding that is presented here. In follow-up analyses that added measures of kinship and time-in-Jordan (just for Syrian refugees) as covariates, overall results were unchanged, *p* values decreased in all analyses, and 5-HTTLPR x Resilience remained significantly associated with PSS (5-HTTLPR x Resilience *p* = 4.26 x 10^−4^, S2 Table).

### COMT

When testing for associations of *COMT* with the six outcome measures, we identified only one significant association (Fig 1, Model 3), which was an interaction between *COMT* and resilience with post-traumatic stress symptoms (CRIES-8) (*COMT* x Resilience *p* = 6.2 x 10^−4^, S1 Table). Plots of *COMT*, CRIES-8, and resilience indicate that participants reported an increase in post-traumatic stress symptoms over time, with the exception of Val homozygotes with high resilience who had stable levels of post-traumatic stress symptoms over time (Fig 2C). Thus, there was a GxE interaction such that the Val allele in a homozygous state appeared protective against rising post-traumatic stress symptoms but only in individuals with high resilience levels, i.e. Val homozygotes with low resilience look the same as Met carriers. This result is consistent with previous studies that identified blunted stress reactivity in Val homozygotes that did not vary with trauma exposure [36]. In follow-up analyses that added measures of kinship and time-in-Jordan (just for Syrian refugees) as covariates, overall results were unchanged, *p* values generally stayed the same (decreased in 14/30 analyses, increased in 13/30 analyses, and stayed the same in 3/30 analyses), and *COMT* x Resilience remained significantly associated with CRIES-8 (*COMT* x Resilience *p* = 7.76 x 10^−4^, S2 Table).

## Discussion

There is great variation in how children and adolescents experience and recover from trauma, which is likely underpinned by a combination of genetic factors, environmental experiences, and interactions thereof. We took an integrative approach to test the effects of both genetic variants and psychosocial factors in a longitudinal case:control study of Syrian refugee and Jordanian non-refugee youth who were differentially exposed to war-related trauma.

We identified one direct and two GxE effects of the tested genetic variants with resilience on multiple measures of psychosocial stress; these effects remained significant after strict correction for multiple testing (Fig 1). Specifically, we identified a direct genetic effect of 5-HTTLPR on human insecurity (HI), which captures feelings of fear and insecurity in conflict-affected areas; in our study, HE homozygotes had lower levels of insecurity (Fig 2A). We also identified a GxE interaction of 5-HTTLPR with resilience and the perceived stress scale (PSS), which is a generalized measure of psychosocial stress; again, the HE allele appeared protective (as a homozygote or heterozygote) and was associated with sharper reductions in PSS over time in individuals with high levels of resilience in contrast to LE homozygotes (Fig 2B). In *COMT*, we identified a GxE interaction with resilience in association with post-traumatic stress symptoms (CRIES-8); the Val homozygote was protective in individuals with high resilience and showed no increase in symptoms over time in contrast to individuals carrying the Met allele (Fig 2C). These results are consistent with our predictions that we would identify protective direct and GxE effects with the tested genetic variants that interacted with high levels of resilience in association with improved scores on a subset of the outcome measures focused on psychosocial stress. Interestingly, the GxE results focus on outcomes related to psychosocial and post-traumatic stress.

As expected due to the nature of GxE effects, the protective effect of the genetic variants (the HE allele in 5-HTTLPR and the Val allele in *COMT*) was only evident in the presence of the environmental factor (high resilience), i.e. there was no difference in outcome measure by genotype in individuals with low resilience. Furthermore, all GxE effects only emerged over time, demonstrating the strength of a longitudinal study to reveal the long-term effects of genetics and environment on psychosocial stress.

In the majority of published studies, the LE variant of 5-HTTLPR has been reported as a risk or vulnerability variant that moderates the effect of a stressful environment on worsened mental health outcomes, as originally proposed by Caspi et al. [16]. Subsequent studies of the relationship between 5-HTTLPR and stress have produced mixed results. Some studies confirm the GxE effect of the LE variant, some report no effect of the LE variant, and some report an effect driven by the HE variant [e.g. 64–67]. However, many studies do not type the rs25531 SNP (in contrast to our study), which would result in LE variants with an untyped G at rs25531 being misclassified as HE variants; misclassification of LE variants could create inconsistent results across studies [68, 69]. Meta-analyses have largely failed to replicate the results [19, 70] although combining different types of stressful events may complicate replication efforts in meta-analyses. However, most studies are not longitudinal studies and do not test protective environmental factors, which were critical components of our reported results. Thus, our identification of a protective effect of the HE variant in association with high levels of resilience may reflect the power of a longitudinal study including protective environmental factors combined with more complete typing of the 5-HTTLPR locus.

In *COMT*, both the Val and Met variants have been reported as risk alleles when studying different study populations and different health outcomes [e.g. 31, 33]. Interestingly, both variants have been associated with increased psychotic symptoms, but the Val allele was linked to cannabis use [71, 72] whereas the Met allele was linked to stress exposures [73, 74]. In our study, we report that Val homozygosity appears to be protective against increasing post-traumatic stress symptoms, but only in individuals with high resilience levels and regardless of trauma exposure, which is consistent with previous studies that identified blunted stress reactivity in Val homozygotes that did not vary with trauma exposure [36].

Our study is the first to report protective GxE effects of 5-HTTLPR and *COMT* variants with high levels of resilience, but not with trauma exposure. These results suggest that the 5-HTTLPR HE variant and the *COMT* Val variant may be differential sensitivity variants, as defined by Belsky and Pluess [9, 10], in which carriers are more susceptible and responsive to the effects of resilience. Furthermore, the fact that these GxE effects are seen only with resilience, and not trauma, supports the vantage sensitivity model that posits some individuals are more sensitive to, and better able to benefit from, positive environmental factors [11, 12]. A recent study that included a range of negative and positive daily events, as well as negative and positive outcome measures, explicitly tested the 5-HTTLPR LE variant for differential susceptibility but instead found the opposite, i.e. HE/HE homozygotes showed more reactivity than LE carriers to negative and positive influences on positive emotions but no effect on negative emotions [67]. The authors speculate that their results may differ from earlier studies because they looked at within-person effects on daily events, which is similar to our longitudinal study design of within-person effects (although our scale was one year). Another recent study [75] reported that carriers of the LE variant showed emotional inertia, a sort of emotional inflexibility that might render individuals less sensitive to negative and positive stimuli, which would suggest the LE variant is not a differential susceptibility factor. Both of these studies are consistent with our results, which suggest that the HE and Val variants function as differential susceptibility variants and are more sensitive to, and more able to benefit from, higher levels of resilience resources.

Monoamine transmitters play a key role in stress response and resilience, and 5-HTTLPR and *COMT* are two of the most widely studied monoaminergic genes. It is useful to speculate on how the 5-HTTLPR HE and the *COMT* Val variants might impact susceptibility to environmental factors or stress reactivity. The HE variant has increased activity (relative to the LE variant) that results in higher serotonin reuptake and lower levels of intrasynaptic serotonin [14, 15] and the Val variant has increased activity (relative to the Met variant) that leads to increased neurotransmitter degradation and lower levels of neurotransmitters [26]. Thus, both variants result in reduced available levels of amine neurotransmitters like serotonin, dopamine, and norepinephrine. Interestingly, drugs like selective serotonin reuptake inhibitors (SSRIs) work to treat depression by increasing intrasynaptic levels of serotonin, i.e. the opposite effect of the HE and Val variants. However, the key characteristic of a differential susceptibility variant is that it enables a more variable, or more plastic, response to environmental factors relative to the other allele [9], thus, we could speculate that lowering the baseline level of available amine neurotransmitters (as the HE and Val variants do) allows for more variation, or increased possibilities, in emotional and behavioral responses to trauma.

### Study limitations

Our sample size of 723 individuals is small, particularly for a genetics study. However, the longitudinal study design with repeated measures over one year allowed us to better control for noise when assessing individual trajectories. The follow-up visits, even accounting for participants lost to follow-up, increased our datapoints to 1534. This sample size was further enhanced by the use of multilevel modeling, which is robust to partially missing data and mitigated the effect of participants lost to follow-up. Kinship can also affect genetic associations. Although we only had information on the presence of siblings in the sample population, siblings represent the closest degree of kinship and also capture information on shared environment–our results were unchanged when information on siblings was included in the analyses. Another limitation is that we only investigate two genetic variants (although both variants occur in important candidate genes for response to trauma). We did not identify gene x trauma interactions as previous studies have, but we did identify significant interactions with resilience and both variants. Resilience is not often included in genetic studies of response to trauma and our results suggest that resilience may be as important as trauma exposure in understanding why individuals vary in their response to trauma. Future studies should investigate additional genetic variants in a larger sample size, while maintaining the longitudinal study design. Notwithstanding the small sample size, our outcome measures appear to have an informative range of variation since we identified GxE effects in three of the outcome measures after correction for multiple testing. In a longitudinal study, data collection of a large number of samples from a highly mobile refugee population will always be challenging [76].

## Conclusions

In our study of refugee and non-refugee youth, we identified genetic and environmental factors, and GxE interactions, that were associated with three measures of psychosocial stress—perceived stress (PSS), post-traumatic stress symptoms (CRIES-8), and human insecurity (HI). We identified two GxE interactions with the tested loci, 5-HTTLPR and *COMT*, and resilience in association with perceived stress and post-traumatic stress symptoms, respectively. In both cases, the 5-HTTLPR HE variant and the *COMT* Val variant had a protective effect that only emerged in individuals with high levels of resilience. Our results point to novel interactions between the protective effects of genetic variants and resilience, lending support to ideas of differential susceptibility and altered stress reactivity. Studies that include the full spectrum of negative and positive life experiences are necessary to fully understand why people respond differently to trauma. More work with vulnerable populations is necessary, particularly with programs that build resilience, in order to better understand how to help individuals heal from trauma.

## Supporting information

S1 Table Results of multilevel modeling to test effects of *COMT*, 5-HTTLPR, trauma, and resilience on six mental health and psychosocial stress outcomes. (XLSX)

S2 Table Follow-up multilevel modeling to test effects of *COMT*, 5-HTTLPR, trauma, and resilience on six mental health and psychosocial stress outcomes with kinship and time-in-Jordan covariates. (XLSX)

S3 Table Demographic, psychosocial, and genetic data for each study participant. (CSV)

S4 Table Codebook describing columns in S3 Table. (XLSX)

S1 File Inclusivity in global research. (DOCX)
